# Developing a Predictive Model for Depressive Disorders Using Stacking Ensemble and Naive Bayesian Nomogram: Using Samples Representing South Korea

**DOI:** 10.3389/fpsyt.2021.773290

**Published:** 2022-01-07

**Authors:** Haewon Byeon

**Affiliations:** Department of Medical Big Data, College of Artificial Intelligence Convergence, Inje University, Gimhae, South Korea

**Keywords:** naive Bayesian nomogram, extreme gradient boosting (XGBoost), stacking ensemble, depressive disorders, multiple risk factors, synthetic minority oversampling technique (SMOTE)

## Abstract

This study provided baseline data for preventing depression in female older adults living alone by understanding the degree of their depressive disorders and factors affecting these depressive disorders by analyzing epidemiological survey data representing South Koreans. To achieve the study objective, this study explored the main risk factors of depressive disorders using the stacking ensemble machine technique. Moreover, this study developed a nomogram that could help primary physicians easily interpret high-risk groups of depressive disorders in primary care settings based on the major predictors derived from machine learning. This study analyzed 582 female older adults (≥60 years old) living alone. The depressive disorder, a target variable, was measured using the Korean version of Patient Health Questionnaire-9. This study developed five single predictive models (GBM, Random Forest, Adaboost, SVM, XGBoost) and six stacking ensemble models (GBM + Bayesian regression, RandomForest + Bayesian regression, Adaboost + Bayesian regression, SVM + Bayesian regression, XGBoost + Bayesian regression, GBM + RandomForest + Adaboost + SVM + XGBoost + Bayesian regression) to predict depressive disorders. The naive Bayesian nomogram confirmed that stress perception, subjective health, n-6 fatty acid, n-3 fatty acid, mean hours of sitting per day, and mean daily sleep hours were six major variables related to the depressive disorders of female older adults living alone. Based on the results of this study, it is required to evaluate the multiple risk factors for depression including various measurable factors such as social support.

## Introduction

South Korea has entered the aged society in 2017 at the fastest rate in the world (1). If this trend continues, it is predicted to enter the super-aged society in 2026 (1). Particularly, the number of older adults living alone is rapidly increasing in South Korea. Statistics Korea (2) reported that the increase in the female older adult population, the increased number of the older old (75 years or older), and the increase in the older adults living alone accounted for the increase in the older adults in South Korea over the past 10 years. Over the past 30 years, the proportion of older adults supported by their families while living with adult children or spouses has decreased but the proportion of older adults living alone has increased, which changed the composition of older adult households (2). The proportion of older adults living alone tended to increase (2). The number of older adults living alone was 190,000 in 1990 and increased to 1.35 million in 2017, a seven-fold increase (2). Due to the favor of the nuclear family as moving into the twenty-first century, the function of supporting older adults has been weakened and more older adults want to live in a space independent from their children in South Korea (3). As a result, it is expected that the number of older adults living alone in South Korea will continue to increase in the future.

The increase of older adults living alone draws attention because most of them are socioeconomically vulnerable (4) and 85% of them are women (2). As of 2020, the life expectancy of South Korean is 80.3 years for men and 86.3 years for women (5). As women are expected to live 6 years longer than men, it is expected that the number of female older adults living alone will increase further (5). Another problem associated with the increased number of older adults living alone is the increase in depressive disorders. The national survey by the Korea Institute for Health and Social Affairs (6) showed that 21.1% of older adults (≥65 years) had depressive symptoms. The Health Insurance Review and Assessment Service (7) also reported that 31.2% of the entire patients with depressive disorders (796,364 patients) were older adults (≥65 years). Particularly, older adults living alone are more likely to be more vulnerable to depressive disorders than those living with their families (4). Therefore, it is necessary to screen high-risk groups by identifying factors related to the depressive disorder of older adults living alone based on these results for preventing the occurrence of depressive disorders of older adults living alone.

The results of previous studies (8–11) argued that female older adults living alone tended to be psychologically atrophied and depressed because they became less socially active due to an increase in chronic degenerative diseases (11). It was also reported that female older adults living alone were more likely to be older, struggle against a disease, be less educated, and have more financial difficulties than male older adults living alone (8). These socioeconomic, personal, and environmental characteristics of female older adults living alone suggested that factors related to their depressive disorders were different from older adults living with their families. Nevertheless, most previous studies that have identified the factors related to the depressive disorders of older adults have focused on older adults living with their families (12) and only a few studies have tried to predict the depressive disorders of female older adults.

For the past 10 years, studies in the medical field have continuously tried to identify risk factors for depressive disorders using data mining techniques as well as traditional statistical analysis models (e.g., regression model) (13). Particularly, recent studies (14, 15) have used the stacking ensemble technique that predicts y-class by integrating multiple individual machine learning techniques to overcome the limitations of a single machine learning technique. It is known that stacking ensemble has higher accuracy than a single learning machine technique because it predicts again using a meta model based on the data predicted by individual algorithms (14). However, it was also reported that the prediction of a stacking ensemble model was lower than that of a single machine learning model depending on the type of algorithm of the base model and meta model (16). Consequently, it is necessary to conduct more stacking ensemble based machine learning studies to find the best-performing ensemble based predictive model for predicting depressive disorders.

When developing a model for predicting a disease using machine learning, it is important that medical personnel can understand the results derived from machine learning, in addition to predictive performance such as accuracy. Therefore, developing eXplainable Artificial Intelligence (X-AI) has become an important issue in machine learning research using medical data in recent years. This study enhanced analysis power by applying a naive Bayesian nomogram to the results derived from machine learning and tried to resolve the intrinsic issue of ensemble-based machine learning's black-box approach (the issue of difficulty in interpreting the results while increasing the accuracy of prediction).

When composing a naive Bayesian nomogram using medical data, it is assumed that all features significantly affect y-class (label). However, not all investigated features have a meaningful effect on y-class. For example, a feature may be barely related to y-class and it may act as noise in some times to make it harder to predict y-class. Therefore, it is very important to select features used in the model when constructing a nomogram. This study developed an ensemble-based predictive model and selected variables to be entered into the nomogram based on the importance of the variables derived from the predictive model as a method of choosing features constituting the naive Bayesian nomogram. As far as we are aware, this is the first study using a stacking ensemble machine technique for selecting variables to be included in the nomogram.

This study provided baseline data for preventing depression in female older adults living alone by understanding the degree of their depressive disorders and factors affecting these depressive disorders by analyzing epidemiological survey data representing South Koreans. To achieve the study objective, this study explored the main risk factors of depressive disorders using the stacking ensemble machine technique. Moreover, this study developed a nomogram that could help primary physicians easily interpret high-risk groups of depressive disorders in primary care settings based on the major predictors derived from machine learning.

## Methods

### Data Source

This study analyzed the raw data of the 7th National Health and Nutrition Examination Survey, conducted from 2016 to 2018 under the supervision of the Korea Centers for Disease Control and Prevention. This study used secondary data. The National Health and Nutrition Examination Survey is a national epidemiological data supervised by the Ministry of Health and Welfare and the Korea Centers for Disease Control and Prevention. It was carried out after receiving written consent from all participants and receiving approval (No.1041107-201806-HR-011-01) from the Institutional Review Board of the Korea Centers for Disease Control and Prevention [please refer to (16) for detailed sampling and survey methods]. In summary, the 7th National Health and Nutrition Examination Survey targeted 24,269 people from 13,248 households, and the survey completion rate (participation rate) was 76.7% (*n* = 18,614). The health survey of the National Health and Nutrition Examination Survey consisted of medical history, activity restrictions and quality of life, physical activity, and health behavior. It was conducted by using interviews and self-interviewing method. The physical examination was composed of physical measurement, blood pressure measurement, and blood test. During the survey period, an examination team consisting of doctors and nurses visited the target area using a mobile physical examination vehicle and conducted a 1:1 checkup and health survey. A nutritional survey was performed by having a nutrition surveyor visit the home of the survey subject and conduct an interview using a food intake frequency survey method. This study selected 634 female older adults (≥60 years old) who completed health surveys, blood pressure measurements, physical measurements, and blood tests as primary analysis subjects. Afterward, this study excluded 61 participants who did not respond (missed) to the Korean version of Patient Health Questionnaire-9 (PHQ-9) (17), a standardized depression screening test, and 582 patients were finally analyzed.

### Measurement of Variables

The depressive disorder, a target variable, was measured using the Korean version of PHQ-9 (17). PHQ-9 is a standardized depression screening test, developed by Spitzer et al. (18), and has been used widely to diagnose mental health in primary health care centers. PHQ-9 consists of nine items corresponding to the diagnostic criteria for major depressive disorders in the Diagnostic and Statistical Manual of Mental Disorders (DSM-IV) (i.e., changes in sleep in the last 2 weeks, changes in appetite, anhedonia, feelings of guilt or worthlessness, feeling down, depressed, fatigued, or restlessness, lowered concentration, and frequency of having suicidal thoughts). It is assessed on a 4-point scale (“never,” “several days,” “more than a week,” or “almost every day”), and the sum of scores ranges from 0 to 27. A higher score means a severer depressive disorder. Depressive disorders were defined as 10 or higher PHQ-9 points based on the results of previous studies (19, 20). Choi et al. (21) reported that the specificity of PHQ-9 was 89.9% and the sensitivity of it was 81.1%. In addition, the reliability of PHQ-9 (Cronbach's α) in this study was 0.89.

### Developing a Model to Predict the Depressive Disorders of Female Older Adults Living Alone

The model for predicting the depressive disorders of female older adults living alone was composed of exploring the best predictive factors for depressive disorders using stacking ensemble (step 1) and developing a naive Bayesian nomogram that could predict high-risk groups of depressive disorders based on major variables derived from machine learning (step 2).

#### Exploring the Best Predictive Factors for Depressive Disorders Using Stacking Ensemble: Base Model

**Figure 1:**
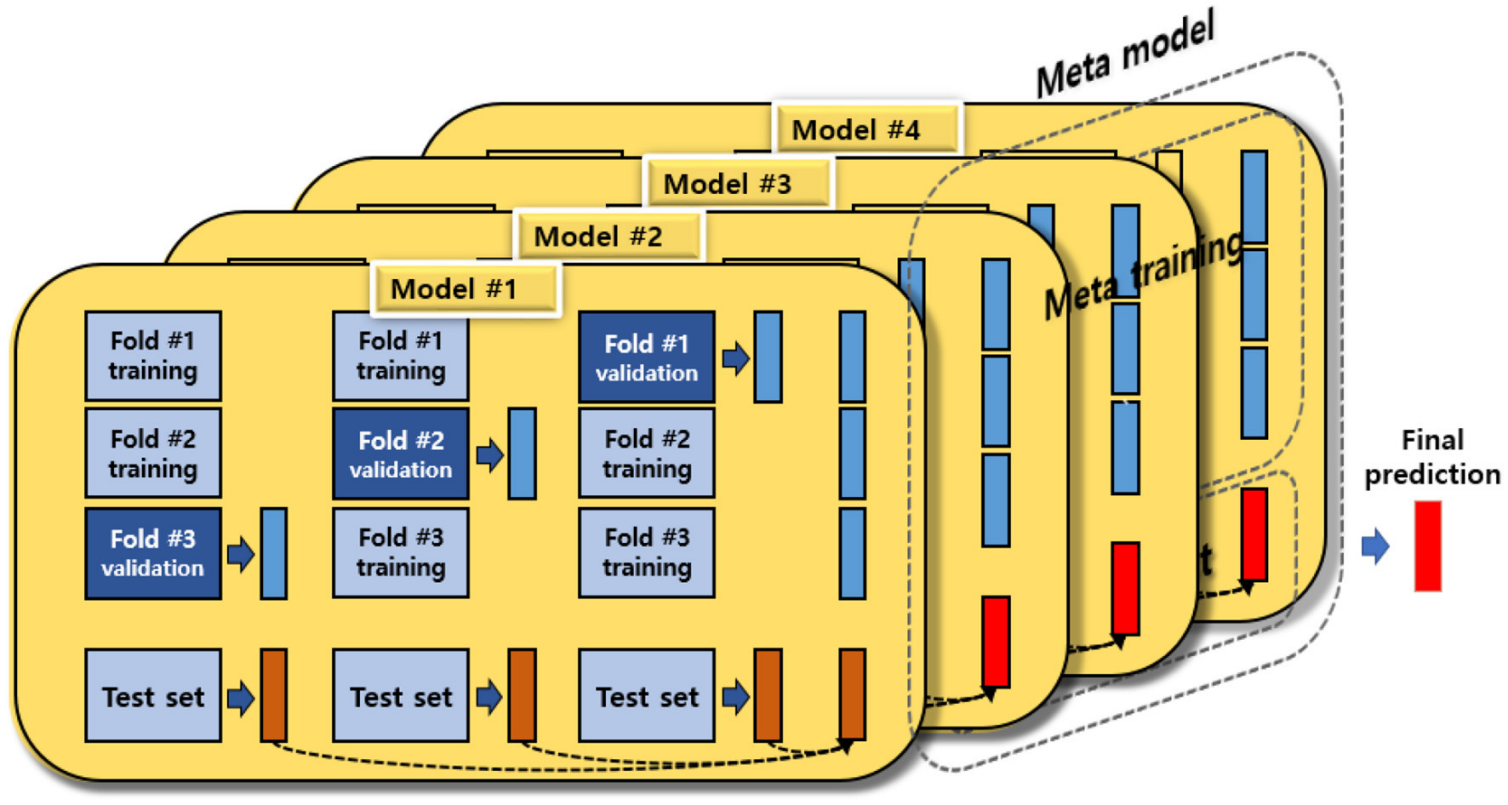
The concept of a stacking ensemble.

This study explored the key variables of depressive disorders in female older adults living alone using the stacking ensemble technique. A number of studies (14, 15, 22) have reported that the stacking ensemble model shows excellent accuracy because it compensates for the overfitting possibility, a disadvantage of a single predictive model. In other words, the goal of the stacking ensemble is to improve generalization capacity, and it has been widely used for classifying and developing predictive models using machine learning. The stacking ensemble creates a new model by combining various different machine learning models as if stacking them in layers (14). It improves the performance of the final model by taking the strengths of each model and complementing the weaknesses of each model while going through two stages [base model (single predictive model and meta model)] (14). The concept of a stacking ensemble is presented in Figure 1.

This stacking ensemble model used GBM, Adaboost, XGBoost, SVM, and random forest as the base model to explore the key factors of depressive disorders in female older adults living alone.

GBM is a type of boosting. Boosting refers to a method to make a more accurate model by gradually improving inaccurate models (23). It creates a model, even if its accuracy is low. Then, it makes up for the weakness of the previous model by applying a large weight to a measurement value with a large error and a small weight to a measurement value with a small error. In this process, it is important to choose an appropriate weight to be applying by measuring the prediction error for each measurement value. GBM determines the weight by using the gradient descent method. If F(x) is assumed as the predicted value of a weak model, the prediction error of each variable can be expressed as the error function L in Equation (1).


(1) $$\begin{array}{l} L\left(y,\;F\;(\text{X})\right)=\overset{N}{\underset{i=1}{\sum }}{\left(y_{i}-F\;(x_{\text{i}})\right)}^{2},\;\forall \;i\in N \end{array}$$

(2) $$\begin{array}{l} \hat{F\;(\text{X})}=\underset{F}{\text{arg}min}\;L\left(F\;(\text{X})\right) \end{array}$$

When the value of Equation (1) shows in Equation (2) is improved *k* = 1, 2, …, *M* times toward the direction of decreasing the prediction error value, the differential slope is defined as the improvement direction of F(x). In the k-th improvement, the direction of the differential slope (*g*_*k*_(*x*)) is defined in Equation (3), and the magnitude of the slope (ρ_*k*_) is defined in Equation (4).


(3) $$\begin{array}{l} g_{k}\left(x_{\text{i}}\right)={\left[\frac{\partial L\left(y_{i},F\;(x_{\text{i}})\right)}{\partial F\left(x_{\text{i}}\right)}|x\right]}_{F\left(x_{\text{i}}\right)=F_{k-1\left(x_{\text{i}}\right)}},\;\forall \;i\in N,\;\forall \;k\in M \end{array}$$

(4) $$\begin{array}{l} \rho_{k}=\underset{\rho }{\text{arg}min}\;L\left({F\;(x_{\text{i}})}_{k-1}-\rho g_{k}\left(x_{\text{i}}\right)\right),\;\forall \;i\in N,\;\forall \;k\in M \end{array}$$

If the multiplication of *g*_*k*_(*x*) and ρ_*k*_ is defined as an improvement amount, the new F(x) is improved according to Equations (5)–(7). The model is improved M times according to this algorithm to construct the final model.


(5) $$\begin{array}{l} f_{k}\;(x_{\text{i}})=\rho_{k}g_{k}\;(x_{\text{i}}),\;\forall \;i\in N,\;\forall \;k\in M \end{array}$$

(6) $$\begin{array}{l} F_{M-1}\;(x_{\text{i}})=\overset{M-1}{\underset{k=0}{\sum }}f_{k}\;(x_{\text{i}}),\;\forall \;i\in N \end{array}$$

(7) $$\begin{array}{l} F_{M}\;(x_{\text{i}})=F_{M-1}\;(x_{\text{i}})+f_{M}\;(x_{i}),\;\forall \;i\in N \end{array}$$

The AdaBoost algorithm is to create a strong classifier by combining weak classifiers. Here, a weak classifier refers to a classifier that has slightly better predictive power than random prediction, and a strong classifier is a classifier that has close to optimal predictive power.

When the AdaBoost algorithm starts, all data have the same probability to be extracted at the beginning of learning. However, it increases the extraction probability of data that are poorly classified and decreases the extraction probability of data that are well-classified. It reiterates learning while adjusting the extraction probability of data. This is explained in Equation (8).


(8) $$\begin{array}{l} D=\left\{\;(x_{1},\;y_{1}),\;\;(x_{2},\;y_{2}),\;\ldots ,\;\;(x_{n},\;y_{n})\right\},\\ \;x_{\text{i}}\in R^{k},\;y_{i}\in \left\{-1,\;+1\right\} \end{array}$$

Let's assume that Equation (8) is a set of training data, and the extraction probability of each dataset is *w*_1_, *w*_2_, …, *w*_*n*_. Initially, the probability of extracting each dataset is the same. That is, $w_{i}^{\left(1\right)}=\frac{1}{n},\;i=1,\;\ldots ,\;n$, where the number of bootstrap samples is *M*, and the classifier derived from each bootstrap sample group is *C*_1_, *C*_2_, …, *and C*_*M*_. Then, the miss-classification error (ϵ_*m*_) of the classifier *C*_*m*_ is calculated as in Equation (9).


(9) $$\begin{array}{l} \epsilon_{m}=\frac{\sum_{i=1}^{n}w_{i}^{\left(m\right)}I\left(C_{m}\left(x_{\text{i}}\right)\neq y_{i}\right)}{\sum_{i=1}^{n}w_{i}},\;m=1,\;\ldots ,\;M \end{array}$$

Here, ϵ_*m*_ is a misclassification rate using the extraction probability of data for each data misclassification by the classifier *C*_*m*_ as a weight. The confidence of the classifier *C*_*m*_ (α_*m*_) is determined as in Equation (10).


(10) $$\begin{array}{l} \alpha_{m}=\frac{1}{2}\ln\frac{1-\epsilon_{m}}{\epsilon_{m}},\;m=1,\;\ldots ,\;M, \end{array}$$

When $w_{i}^{\left(m\right)}$ is the extraction probability of the mth bootstrap sample, the extraction probability of the (m+1)th bootstrap sample ($w_{i}^{\left(m+1\right)}$) is calculated as in Equation (11).


(11) $$\begin{array}{l} w_{i}^{\left(m+1\right)}=\frac{w_{i}^{\left(m\right)}\exp\left(-\alpha_{m}y_{i}C_{m}\left(x_{i}\right)\right)}{Z_{m}} \end{array}$$

Here, *Z*_*m*_ is a normalization constant and satisfies Equation (12).


(12) $$\begin{array}{l} Z_{m}=\overset{n}{\underset{i=1}{\sum }}w_{i}^{\left(m\right)}\exp\left(-\alpha_{m}y_{i}C_{m}\left(x_{i}\right)\right) \end{array}$$

The final classifier (C^*^(***x***′)) created by combining the M classifiers generated for the new test data ($x_{\text{i}}^{\prime }\in R^{k}$) is as Equation (13).


(13) $$\begin{array}{l} C^{*}(x^{\prime })=sign(\overset{M}{\underset{m=1}{\sum }}\alpha_{m}C_{m}(x^{\prime })) \end{array}$$

Here,

C^*^(***x***′) is a weighted bound formula, obtained by reflecting weights as much as the importance of each classifier (α_*m*_).

XGBoost is an advanced version of gradient boosting. The main goal is to increase speed and the efficiency of competition. XGBoost basically uses a technique called boosting, which increases accuracy by binding weak classifiers. At this time, XGBoost tends to fill in the missing values and refers to extreme slope improvement.

The probability of predicting Z correctly for learner A is as follows.


$$\begin{array}{l} Z=A\;(x)+Error\;\left(A\right) \end{array}$$

When it is assumed that there is learner B that can classify “Error” precisely (Error > Error 2),


$$\begin{array}{l} Error=B\;(x)+\;Error\;2\;\left(B\right) \end{array}$$

When it is assumed that there is learner C that can classify “Error 2” more precisely (Error 2 > Error 3),


$$\begin{array}{l} Error\;2=C\;(x)+\;Error\;3\;\left(C\right) \end{array}$$

When (B) and (C) are applied to (A), Equation (14) is derived.


(14) $$\begin{array}{l} Z=A\;(x)+B\;(x)+C\;(x)+Error\;3 \end{array}$$

Although the accuracy of it is higher than when learner A is used alone, classifiers A, B, and C have different performances. Therefore, since they all have the same ratio (1^*^A + 1^*^B + 1^*^C), it may increase errors by interfering with random x. In this case, weights are applied in front of each model, and the optimal weight is found by using machine learning, which is presented in Equation (15). The model of Equation (15) becomes a classifier with better performance than the model of Equation (14) (Error 3 > Error 4).


(15) $$\begin{array}{l} Z=\alpha^{*}A\;(x)+\beta^{*}B\;(x)+\gamma^{*}C\;(x)+Error\;4 \end{array}$$

SVM predicts using a supporting vector that supports decision-making. The strength of SVM is that it can find a divisional plane even if data are overlapped by expanding the dimension of predictor variables using various kernel functions, unlike linear regression. Moreover, it tends to overfit less because it uses only minimum data supporting decision-making. SVM models in a direction that minimizes the sum of reciprocals of the distance between prediction errors, divisional planes, and supporting vectors.

If the predictor variable is ***x***_**i**_(*i* = 1, 2, 3, ⋯ , *N*), when *y*_*i*_(*i* = 1, 2, 3, ⋯ , *N*) is given for each data, the coefficient β is obtained when Equation (16) is minimal.


(16) $$\begin{array}{l} \min C\overset{N}{\underset{i=1}{\sum }}\left(\xi_{i}+\hat{\xi_{i}}\right)+\frac{1}{2}{∥\beta ∥}^{2} \end{array}$$

ϵ_*i*_ in the right term of Equations (17) and (18) indicates the tolerance range. If the difference between the actual value and the predicted value is less than ϵ_*i*_, the error value is 0, and if it is greater than ϵ_*i*_, the error value is imposed. Slack variables, ξ_*i*_ and $\hat{\xi_{i}}$, indicate how far the predicted value deviates from the sum of the measurement and the error tolerance (ϵ_*i*_), and has a value greater than 0 depending on Equations (19) and (20). The right term in Equation (16) maximizes the distance between the coefficient β and the support vector by minimizing the magnitude of the coefficient.


(17) $$\begin{array}{l} s.t.\;t_{i}\leq F\;(x_{\text{i}})+\epsilon_{i}+\xi_{i},\;\forall \;i\in N \end{array}$$

(18) $$\begin{array}{l} t_{i}\geq F\;(x_{\text{i}})+\epsilon_{i}+\hat{\xi_{i}},\;\forall \;i\in N \end{array}$$

(19) $$\begin{array}{l} \xi_{i}\geq 0,\;\forall \;i\in N \end{array}$$

(20) $$\begin{array}{l} \hat{\xi_{i}}\geq 0,\;\forall \;i\in N \end{array}$$

Random forest is a supervised learning model (24). It consists of a number of decision trees capable of both classification and regression (24). It is designed to overcome the overfitting problem of decision trees (24). It reiterates random sampling of predictors and observations to create multiple decision trees. After obtaining prediction categories from numerous decision trees, it determines the final category prediction using a majority vote method. It can iteratively make independent decision trees by giving randomness to the decision tree formation, and it is possible to reduce prediction error using this method. A bootstrapping technique is used for selecting predictors and observations randomly.

#### Meta Model

**Figure 2:**
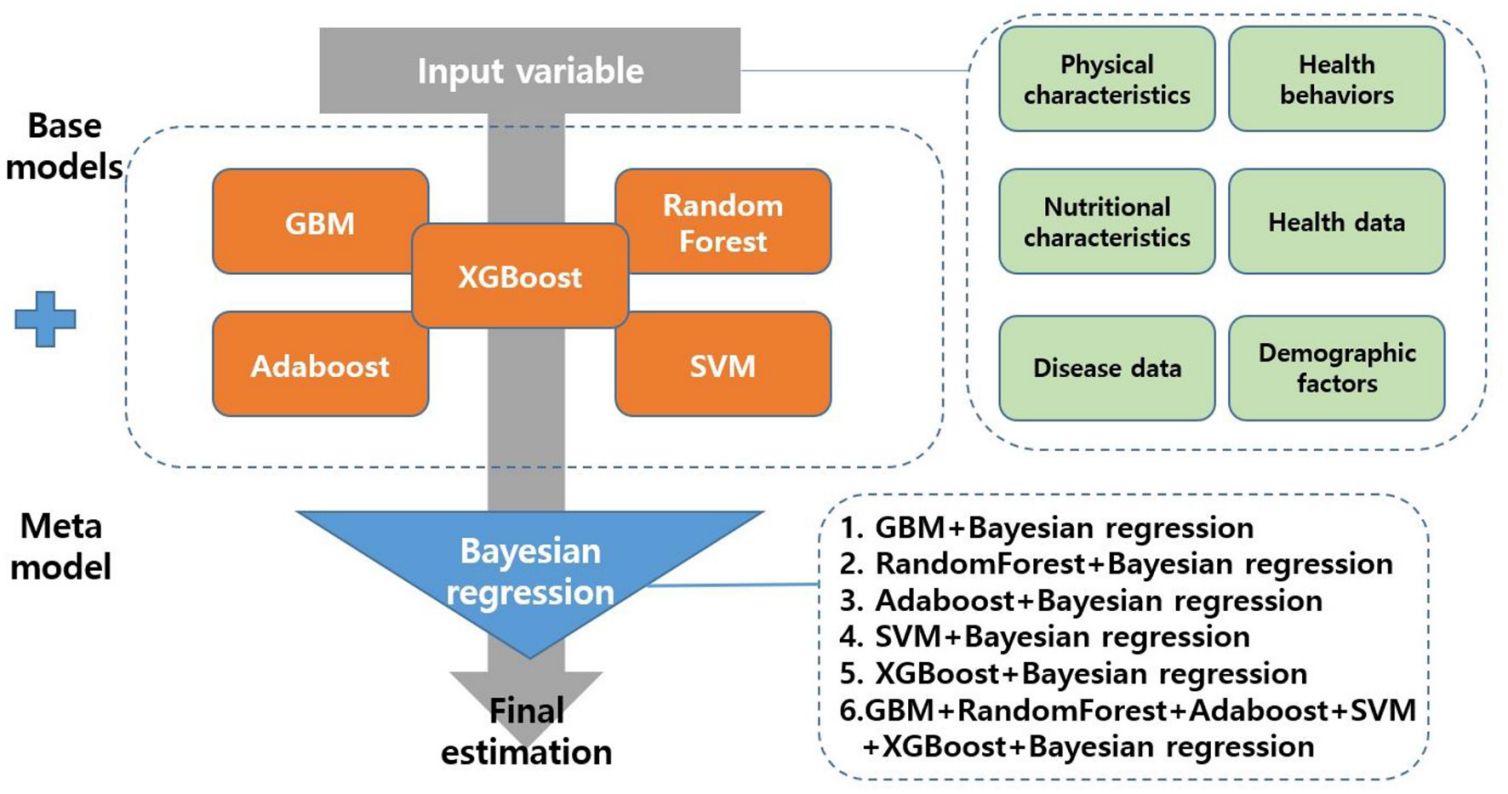
Process flow diagram for depressive disorders of female older adults living alone.

This study used Bayesian regression for the meta model. The regression algorithm increases the reliability of the base model while maximizing the stability of the model (25). Previous studies (25, 26) also reported that the predictive performance such as accuracy was improved compared to a single predictive model, when regression was used for the meta model. Therefore, this study used the regression algorithm for the meta model. Finally, this study developed five single predictive models (GBM, RandomForest, Adaboost, SVM, XGBoost) and six stacking ensemble models (GBM + Bayesian regression, RandomForest + Bayesian regression, Adaboost + Bayesian regression, SVM + Bayesian regression, XGBoost + Bayesian regression, GBM + RandomForest + Adaboost + SVM + XGBoost + Bayesian regression) to predict depressive disorders in female older adults living alone (Figure 2).

#### Testing the Predictive Performance of Stacking Ensemble Model

Since the number of people with a disease is smaller than that of those without a disease, a binary classifier is generally likely to cause a class imbalance issue. This study also found that the ratio of older adults without depression to those with depression was 87.5:12.5 and showed an imbalance problem. Therefore, this study resolved the imbalance issue of binary datasets using the synthetic minority oversampling technique (SMOTE) method. SMOTE is an oversampling technique that generates synthesis data using the k-nearest neighbor algorithm. In other words, it means a method of generating new data between a near minority class and random minority class data. SMOTE first selects random data from a minority class and then sets a k-nearest neighbor in the next data. In the next step, synthesis data are generated between the random data and the random k-nearest neighbor. This procedure is repeated until the minority class and the majority class have the same ratio. The procedure for performing SMOTE is presented in Table 1.

**Table 1:** The procedure for performing SMOTE.

**SMOTE algorithm**	
Step 1	It calculates K-nearest neighbors for *x*_*i*_, a sample, belonging to a minority class.
Step 2	It randomly selects one neighbor $\left(\hat{x_{i}}\right)$ among the K-nearest neighbors, obtained in step 1, to calculate the Euclidian distance from the sample *x*_*i*_.
Step 3	It generates a new sample (*x*_*new*_) by multiplying the Euclidian distance obtained in step 2 with a random value between 0 and 1 and then adding it to the original sample.
	$x_{new}=x_{i}+\left(\hat{x_{i}}-x_{i}\right)\times \delta ,\;\delta \in \left[0,\;1\right]\;$
Step 4	The new sample (*x*_*new*_) generated in step 3 is added to the training data.

**Figure 3:**
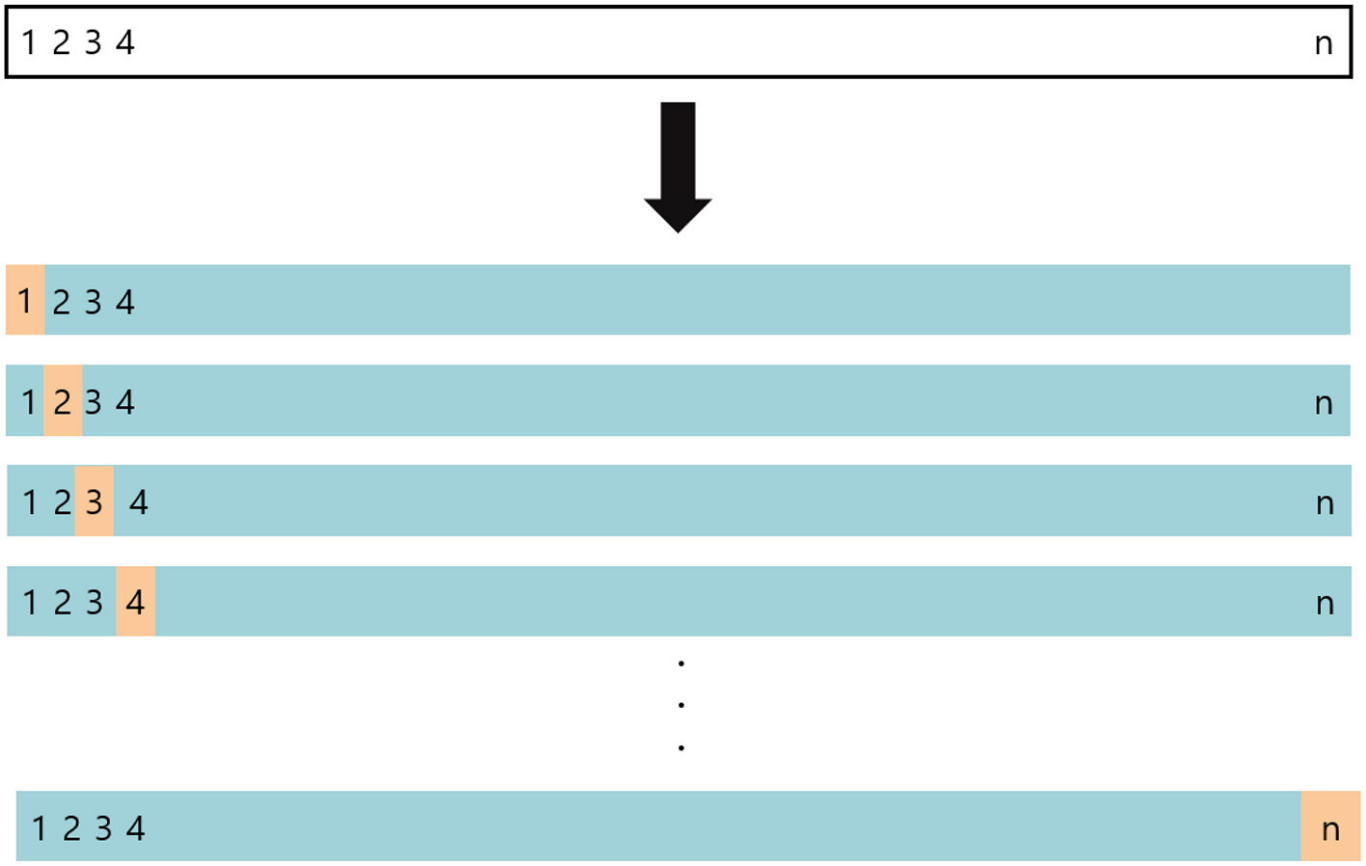
The concept of the LOOCV method.

The predictive performance of the developed 11 machine learning models was tested using the leave-one-out cross-validation (LOOCV) method. LOOCV is a validation method that complements the shortcoming of the validation set approach, which is likely to produce different results when a different random set is drawn. LOOCV produced models N times. When creating each model, only one sample was excluded and the test set performance of it was calculated using the excluded samples. Afterward, it calculates the mean of N performance (Figure 3). When each sample is used as a test set, the mean squared error (MSE) of ith sample is MSE_i_: $MSE_{i}={\left(y_{2}-{ŷ}_{2}\right)}^{2}$ (Equation 21).


(21) $$\begin{array}{l} CV_{\left(n\right)}=\frac{1}{n}\overset{n}{\underset{i=1}{\sum }}MSE_{i} \end{array}$$

This study used F1-score, accuracy, recall, and precision as indices to evaluate predictive performance. The equation of each evaluation index is presented below.

True positive (TP) = It is actually a depressive disorder r, and the predicted outcome is a depressive disorder.
False negative = It is actually a depressive disorder, but the predicted outcome is normal.
False positive = It is actually normal, and the predicted outcome is a depressive disorder.
True negative = It is actually normal, and the predicted outcome is normal.
Recall = TP/(TP + FN)
Precision = TP/TP + FP
Accuracy = TP + TN/TP + TN + FP + FN
F1-score = 2^*^(precison^*^recall)/(precision+recall)

This study assumed that the model with the highest F1-score was the model with the best accuracy in predicting depressive disorders of female older adults living alone. If the F1-scores of two models are the same, the model with the higher recall was defined as a better model.

#### Developing Nomogram Based on Naive Bayesian Technique

**Figure 4:**
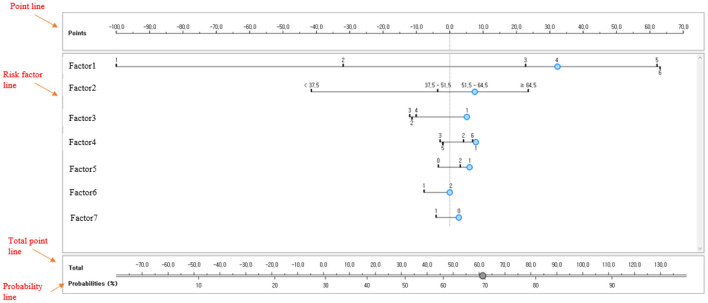
Example of a naive Bayesian nomogram.

This study developed a naive Bayesian nomogram with reference to Možina et al. (27) so that clinicians could easily interpret the prediction (predicted probabilities) using the major variables derived from the final model for predicting depressive disorders. In general, a nomogram consists of 4 types of lines. First, a point line is presented. The score line is a line placed at the top of the nomogram to derive a score corresponding to the class of each risk factor. In the naive Bayesian nomogram, positive numbers are interpreted as risk factors and negative numbers are understood as preventive factors. Second, a risk factor line is presented. The number of risk factor lines is equal to the number of risk factors affecting depressive disorder. Third, the total point line refers to the sum of the scores of individual risk factors and is located at the bottom of the nomogram. Fourth, a probability line is presented. The probability line is the final sum of the nomogram scores calculated by adding up multiple risk factors. It is placed at the bottom of the nomogram to calculate the occurrence probability of depressive disorders in female older adults living alone. Figure 4 shows an example of a naive Bayesian nomogram.

This study used the following notation to describe the content for convenience.

*A* = {*a*_1_, *a*_2_, …, *a*_*m*_}: A set of features used to represent a dataset
*C* = {0, 1}: The label of a class; where 0 denotes another class and 1 denotes the target class.
*D* = {*d*_1_, *d*_2_, …, *d*_*n*_}: A set of patient data
*d*_*i*_ = (υ_*i*1_, υ_*i*2_, …, υ_*im*_, *c*_*i*_): **i**th data.
υ_*ij*_ refers to the value of a feature (*a*_*j*_)
*c*_*i*_ represents a class.
*N*_*j*_: *j*-th generated nomogram
*E*_*N*_*j*__ (*d*_*i*_): The value evaluate the degree of data (*d*_*i*_) belonging to the target class by using nomogram *N*_*j*_
(*d*_(1)_, *d*_(2)_, …, *d*_(*n*)_): A sequence of a data set *D* in the increasing order of *E*_*N*_*j*__ (*d*_*i*_)
*D*_*P*_ = {*d*_*k*_ | *c*_*k*_ = *c, d*_*k*_ ∈ *D*}: A subset of *D* belonging to the target class *c*
*D*_*N*_ = {*d*_*k*_ | *c*_*k*_ ≠ *c, d*_*k*_ ∈ *D*}: A subset of *D* not belonging to the target class *c*
*n*_*P*_ = |*D*_*P*_|: *Number of data in D*_*P*_*n*_*N*_ = |*D*_*N*_|: *Number of data in D*_*N*_
$p_{j}^{P}\left(\upsilon \right)=\frac{\left\{d_{i}|a_{i}^{j}=\upsilon ,\;d_{i}\in D_{P}\;\right\}}{n_{P}}:\;$Relative frequency of data in which the jth features in Dp has a value υ
$p_{j}^{N}\left(\upsilon \right)=\frac{\left\{d_{i}|a_{i}^{j}=\upsilon ,\;d_{i}\in D_{N}\;\right\}}{n_{N}}:\;$ Relative frequency of data in which the jth features in DN has a value υ

The characteristics of the naïve Bayesian classifier was used to construct a nomogram using the naive Bayesian technique. The naive Bayesian classifier determines the probability of a specific class by applying the Bayesian theorem under the assumption that data features or events are independent of each other. Using the assumption of feature independence, the posterior probability (P(c|X)), which is the probability that an entity X =(a_1,a_2,…,a_m) belongs to class c, is calculated as follows.


(22) $$\begin{array}{l} p\left(c|X\right)=\frac{P\left(a_{1},a_{2},\ldots ,a_{m}|c\right)P\left(c\right)}{P\left(X\right)}=\frac{P\left(c\right)\prod_{i}P\left(a_{i}|c\right)}{P\left(X\right)} \end{array}$$

A nomogram can also be considered as a model for evaluating how well an entity fits a particular class. When c is the target class of the nomogram and c is a class other than c, P(c|X) indicates the probability that an entity X does not belong to class c. The odds ratio (Odds) for these two probabilities can be expressed as follows using Equation (23).


(23) $$\begin{array}{l} Odds=\frac{P\left(c|X\right)}{P\left(¯c|X\right)}=\frac{P\left(c\right)\prod_{i}P\left(a_{i}|c\right)}{P\left(¯c\right)\prod_{i}P\left(a_{i}|¯c\;\right)} \end{array}$$

log *it* is defined as the logarithm of *Odds*.

The logit for (*c*|) can be expressed as the following.


(24) $$\begin{array}{l} \log it\;P\left(c|X\right)=logit\;P\left(c\right)+\underset{i}{\sum }\log\frac{P\left(a_{i}|c\right)}{P\left(a_{i}|¯c\right)}\\ \;=logit\;P\left(c\right)+\underset{i}{\sum }\log OR\;(a_{i}) \end{array}$$

The above Equation (24) shows that the logit value of *P*(*c*|*X*) can be expressed as the sum of the log *OR* (*a*_*i*_) of each feature value. Since the final probability value can be expressed as the sum of the evaluation values of each feature value, this property is similar to the method of interpreting the nomogram. Consequently, by using the above properties of the naive Bayesian classifier, a nomogram can be generated through the following process.

1. For the given data D, calculate the relative frequencies ($p_{i}^{P}\left(\upsilon_{j}\right)$ and $p_{i}^{N}\left(\upsilon_{j}\right)$) of the value (υ_*j*_) of each feature (*a*_*i*_) in the target class *c* and the relative class *c*.
2. Calculate the log *OR* for the value (υ_*j*_) of each feature (*a*_*i*_).
  $$\begin{array}{l} \log OR\;(\upsilon_{j})=log\frac{p_{i}^{P}\left(\upsilon_{j}\right)}{p_{i}^{N}\left(\upsilon_{j}\right)} \end{array}$$
3. Evaluation Function (*E*(*d*_*i*_)) for Data di Is Defined by $\underset{j}{\sum }logOR\;(\upsilon_{ij})$, While Treating *logOR* (υ_*j*_) as the score for the corresponding feature *a*_*i*_.
  (25) $$\begin{array}{l} E\left(d_{i}\right)=\underset{a_{j}\in SAT}{\sum }\log OR\left(\upsilon_{ij}\right),\;\log OR\;(\upsilon_{ij})=\log_{10}\frac{p_{j}^{P}\left(\upsilon_{ij}\right)}{p_{j}^{N}\left(\upsilon_{ij}\right)} \end{array}$$
4. Find the maximum (max) and minimum (min) for all possible feature value combinations.
5. Determine the probability (*p*(*c*|*d*_*i*_)) for the interval [min, max] that the data *d*_*i*_ belongs to the target class *c* using the following equation.
  (26) $$\begin{array}{l} p\left(c|X\right)={\left[1+e^{-\log\;itP\left(c\right)-E\left(d_{i}\right)}\right]}^{-1} \end{array}$$
6. Prepare a graph for the nomogram using the logOR for each feature and the probability for the interval (min, max).

The accuracy of the final nomogram was checked using general accuracy, the area under the curve (AUC), and calibration plot, which visually confirms the agreement between predicted probability and observed probability in the nomogram, using the LOOCV.

## Results

### The Data, Measurement Unit of Female Older Adults Living Alone in South Korea

The mean age of the 582 subjects was 72.9 ± 6.20 years, and the prevalence of depressive disorders was 12.5% (Table 2). The majority of the subjects experienced drinking during their lifetime (62.8%), did not experience binge drinking in the past year (92.7%), were not smokers (90.6%), had a mean monthly household income of less than KRW 1.5 million (88.6%), tried to lose weight in the past year (30.4%), were older adults who did not engage in moderate-intensity physical activity (97.8%), were middle school graduates or below (86.4%), were unemployed (69.0%), had normal weight (39.1%), ate breakfast 5–7 times per week on average for the past year (88.9%), had ordinary subjective health (46.6%), did not have diabetes (45.3%), did not have hypertension (66.3%), did not have hypertriglyceridemia (59.3%), did not have hypercholesterolemia (87.4%), and slept 7–8 h per day (27.5%). The subjects sat 9.0 ± 4.0 h per day on average. Moreover, they ate 1.35 ± 1.8 g of n-3 fatty acid and 5.47 ± 6.7 g of n-6 fatty acid. Since only one older adult had class 3 obesity (0.2%), “class 3 obesity” was reclassified as “class 2 obesity or high” in the development of the predictive model.

**Table 2:** Data, measurement unit of female older adults living alone in South Korea, mean ± SD/*n* (%).

**Characteristics**	***n* (%)**
Age	72.9 ± 6.20
**Alcohol consumption**	
No	214 (37.2)
Yes	364 (62.8)
**Binge drinking**	
No	535 (92.7)
Yes	42 (7.3)
**Smoking**	
No	552 (90.6)
Yes	54 (9.4)
**Whether or not to receive national basic livelihood security**	
No	456 (78.5)
Yes	125 (21.5)
**Perception of subjective body type**	
Very thin	43 (7.4)
Slightly skinny	55 (9.5)
Average	226 (39.1)
Slightly obese	192 (33.2)
Very obese	62 (10.7)
**Control weight over the past year**	
Try to lose weight	176 (30.4)
Try to maintain weight	72 (12.5)
Try to gain weight	33 (5.7)
Never tried to control weight	297 (51.4)
**Monthly mean household income**	
<KRW 1.5 million	514 (88.6)
≥KRW 1.5 million	66 (11.4)
**Education level**	
Middle school graduation or below	503 (86.4)
High school graduation or above	78 (13.4)
**Level of stress awareness**	
I feel stressed very much	30 (5.2)
I feel stressed a lot	82 (14.2)
I feel stressed a little	246 (42.7)
I hardly feel stressed	218 (37.8)
**Obesity by body mass index (BMI, kg/m** ^**2**^ **)**	
Underweight (<18.5 kg/m^2^)	14 (2.5)
Normal weight (≥18.5 kg/m^2^ and <23 kg/m^2^)	228 (40.1)
Pre-obesity stage (≥23 kg/m^2^ and <25 kg/m^2^)	193 (33.9)
Stage 1 obesity (≥25 kg/m^2^ and <30 kg/m^2^)	104 (18.3)
Stage 2 obesity (≥30 kg/m^2^ and <35 kg/m^2^)	29 (5.1)
Stage 3 obesity (≥35 kg/m^2^)	1 (0.2)
**Mean frequency of having breakfast per week for the past year**	
5–7 times per week	480 (88.9)
3–4 times per week	25 (4.6)
1–2 times per week	13 (2.4)
Rarely	22 (4.1)
**Moderate-intensity physical activity**	
Yes	13 (2.2)
No	569 (97.8)
**Subjective health status**	
Good	79 (13.6)
Okay	271 (46.6)
Bad	232 (39.9)
**Hypertension**	
Normal	94 (16.2)
Prehypertension	102 (17.5)
Hypertension	386 (66.3)
**Diabetes**	
Normal	243 (45.3)
Impaired fasting glucose	148 (27.6)
Diabetes	145 (27.1)
**Hypertriglyceridemia**	
No	423 (87.4)
Yes	61 (12.6)
**Hypercholesterolemia**	
No	318 (59.3)
Yes	218 (40.7)
Waist Circumference (cm)	84.6 ± 9.8
N-3 fatty acid intake per day (g)	1.35 ± 1.8
N-6 fatty acid intake per day (g)	5.47 ± 6.7
Vitamin c intake per day(g)	50.1 ± 57.6
Energy intake per day (Kcal)	1,416.8 ± 603.9
Water intake per day (g)	693.5 ± 497.7
Protein intake per day (g)	45.6 ± 25.0
Cholesterol intake per day (g)	113.6 ± 174.9
Carbohydrate intake per day (g)	254.6 ± 109.0
Calcium intake (mg)	386.6 ± 264.8
Vitamin A intake per day (ug)	432.9 ± 483.4
Usual hours of sitting per day	9.0 ± 4.0
**Usual hours of sleep per day**	
<5	49 (8.5)
5–6	72 (12.4)
6–7	108 (18.7)
7–8	159 (27.5)
8–9	116 (20.0)
>9	75 (13.0)
**Depressive disorders**	
No	509 (87.5)
Yes	73 (12.5)

### Comparing the Accuracy of Models Predicting the Depressive Disorders of Female Older Adults Living Alone

**Figure 5:**
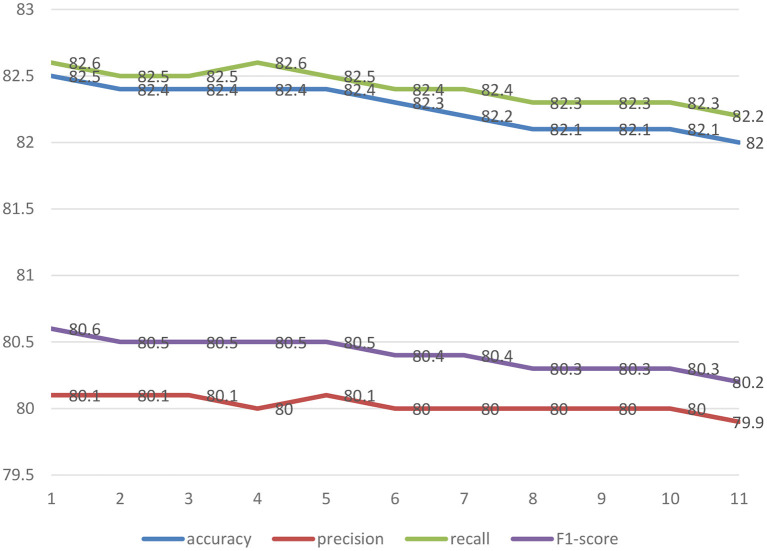
The comparison of 11 machine learning models (accuracy, precision, recall, F1-score) for predicting the depressive disorders of female older adults living alone.

Figure 5 shows the comparison of 11 machine learning models (accuracy, precision, recall, F1-score) for predicting the depressive disorders of female older adults living alone. The analysis results showed that the prediction performance of “GBM + RandomForest + Adaboost + SVM + XGBoost + Bayesian regression” was the best (accuracy = 82.52%, precision = 80.13%, recall = 82.60%, and F1-score = 80.62%).

1 = GBM + RandomForest + Adaboost + SVM + XGBoost + Bayesian regression; 2 = GBM + Bayesian regression 3. Adaboost + Bayesian regression; 4 = XGBoost + Bayesian regression; 5 = RandomForest + Bayesian regression; 6 = SVM + Bayesian regression; 7 = GBM; 8 = Adaboost; 9 = RandomForest; 10 = XGBoost; 11 = SVM.

### Exploring the Major Predictors of the Depressive Disorders of Female Older Adults Living Alone

**Figure 6:**
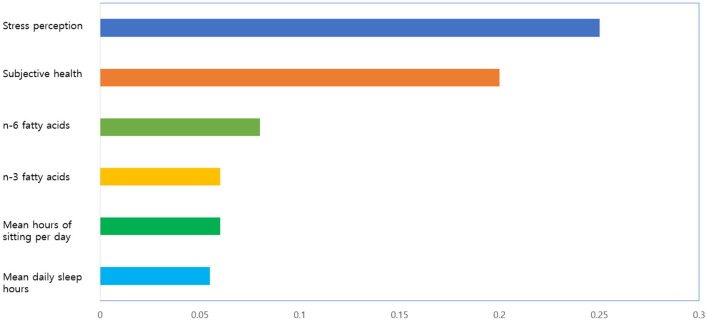
The feature importance (best 6) of final model.

Figure 6 presents the feature importance of GBM + RandomForest + Adaboost + SVM + XGBoost + Bayesian regression model, the final model. This study calculated the importance of a variable using mean decrease in impurity (MDI) Importance. MDI is an index for calculating the importance of a variable, built in as a default of scikit-learn. When each variable is split, it defines the mean of the decrease of impurity as the importance. The function is as shown in Equation (27). The decrease of impurity is calculated while considering the number of observations in each node. A higher value indicates that the importance is higher.


(27) $$\begin{array}{l} \Delta i\left(t\right)=i\left(t\right)-\frac{N_{tl}}{N_{t}}i\left(t_{l}\right)-\frac{N_{tr}}{N_{t}}i\left(t_{r}\right) \end{array}$$

*i* (*t*): impurity of node t (entropy, gini index, variance, …)

*N*_*t*_: number of observations at node t

The model confirmed that stress perception, subjective health, n-6 fatty acids, n-3 fatty acids, mean hours of sitting per day, and mean daily sleep hours were six major variables related to the depressive disorders of female older adults living alone. Among them, stress perception was the most important factor in the final model.

### Development of a Nomogram for Predicting the Depressive Disorders of Female Older Adults Living Alone

**Figure 7:**
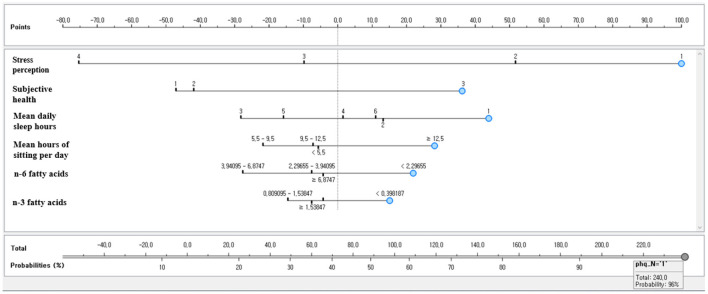
The naive Bayesian nomogram for predicting the depressive disorders of female older adults living alone in South Korea.

Figure 7 shows the naive Bayesian nomogram for predicting the depressive disorders of female older adults living alone in South Korea. Stress perception level showed the highest influence among the risk factors of depressive disorders for female older adults living alone. Female older adults living alone who perceived stress a lot were most vulnerable to depressive disorders. For example, the developed Bayesian nomogram predicted that female older adults living alone who perceived a lot of stress, perceived that their subjective health was bad, slept <5 h per day on average, sat 12.5 h or more per day on average, ate <2.29 g of N6 fatty acid, and ate <0.39 g of N3 fatty acid had the 96% probability of having a depressive disorder (Figure 6).

**Figure 8:**
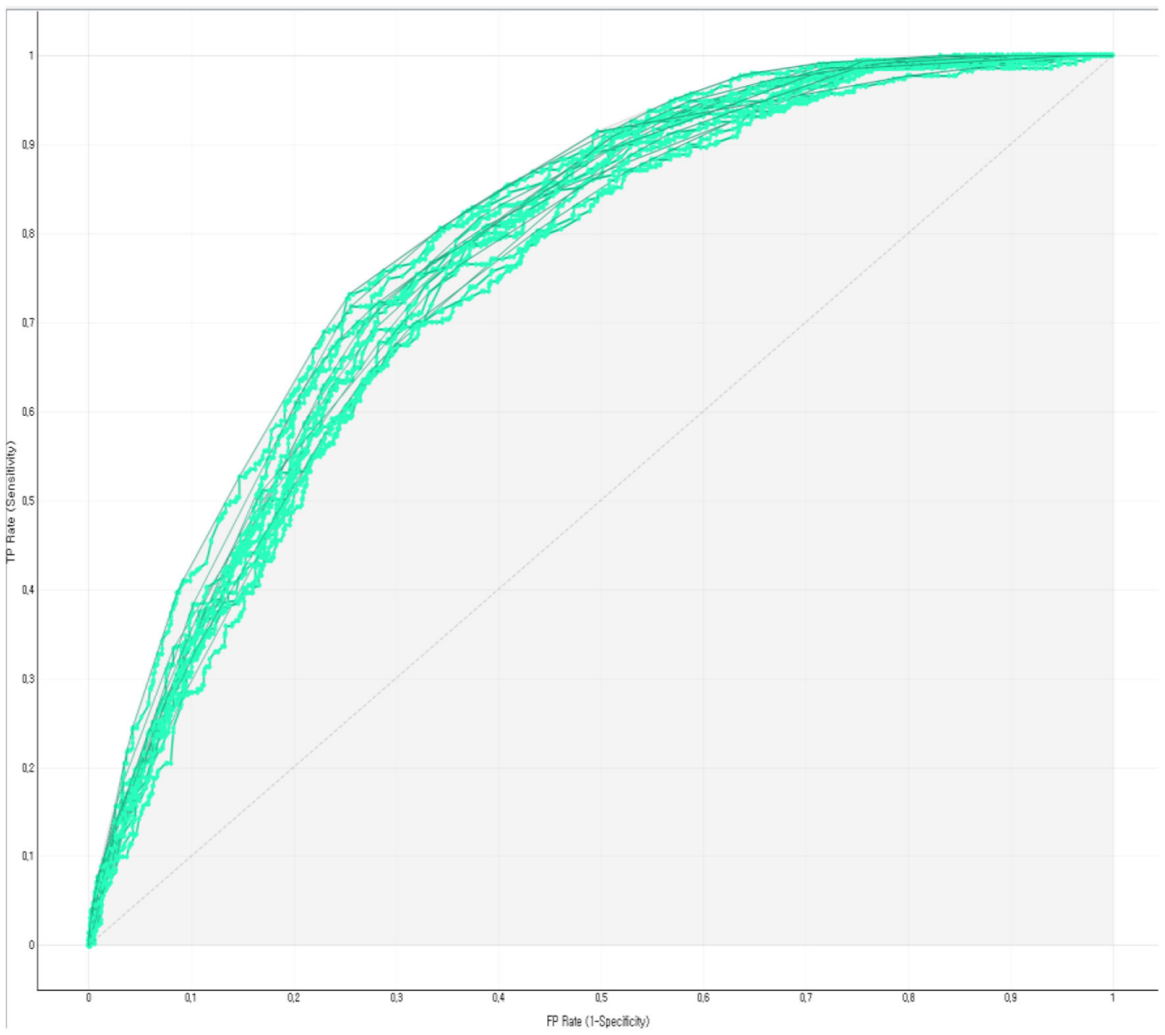
ROC of the Bayesian nomogram for predicting the depressive disorders of female older adults living alone.

**Figure 9:**
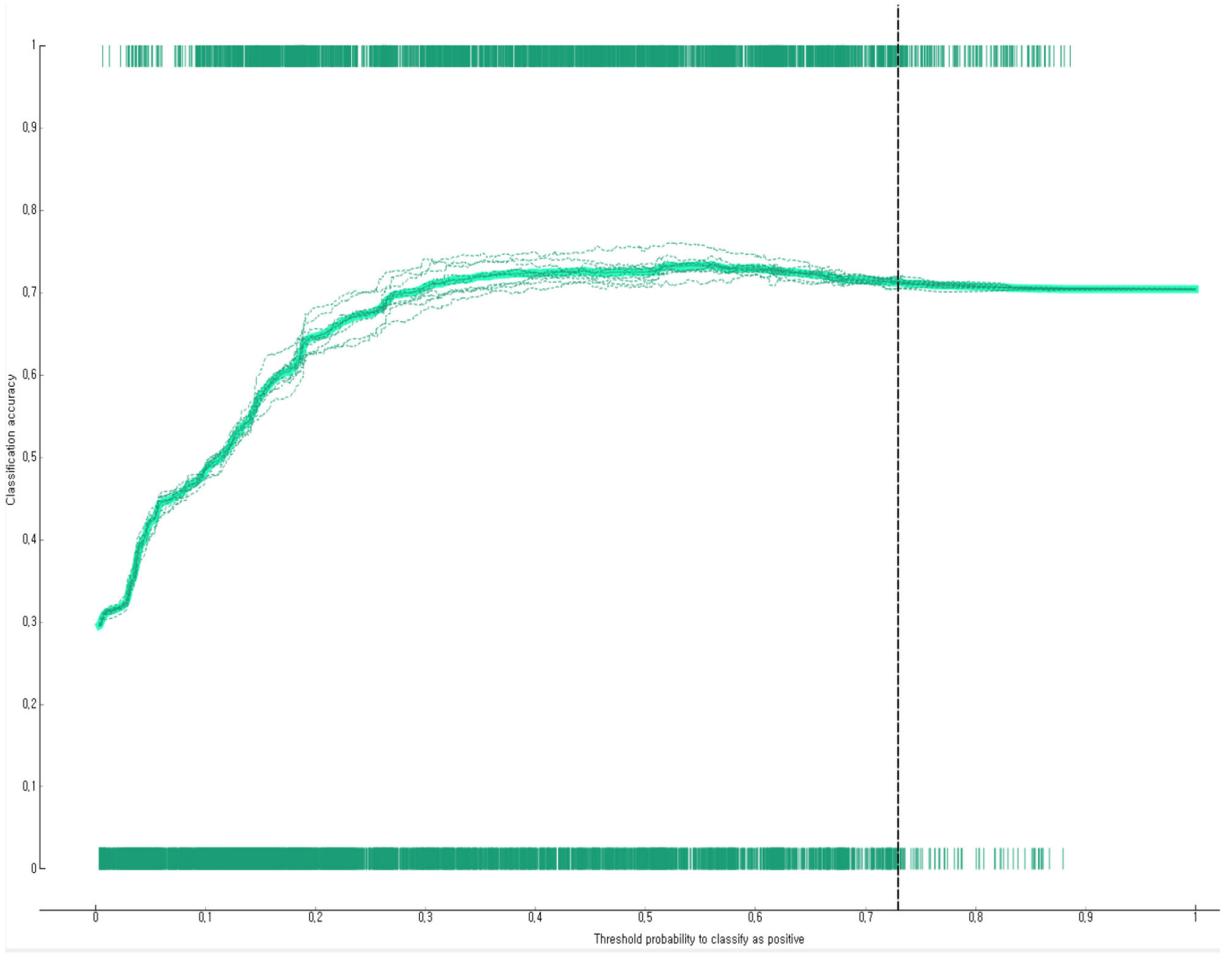
Classification accuracy of the Bayesian nomogram for predicting the depressive disorders of female older adults living alone.

**Figure 10:**
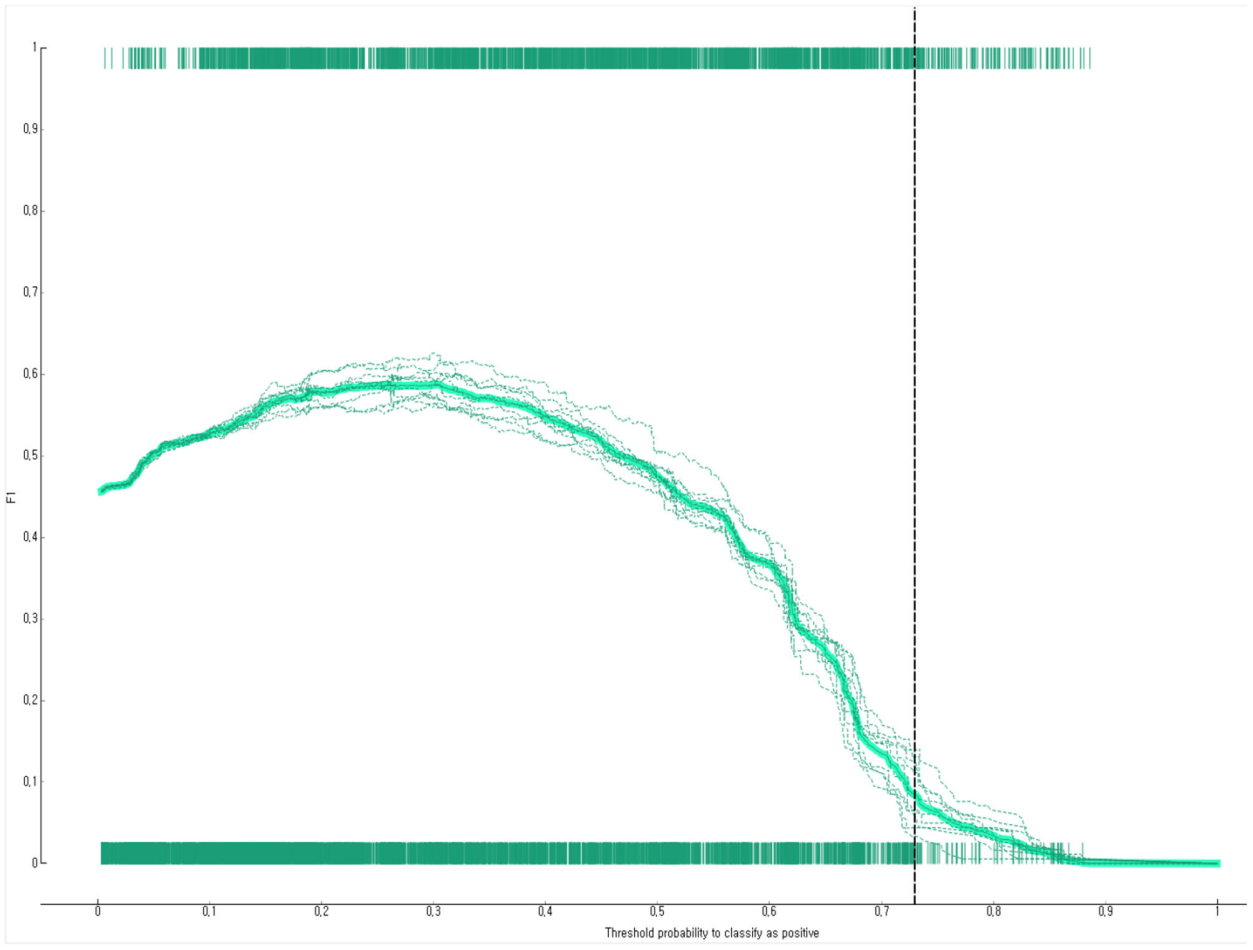
F1-score of the Bayesian nomogram for predicting the depressive disorders of female older adults living alone.

**Figure 11:**
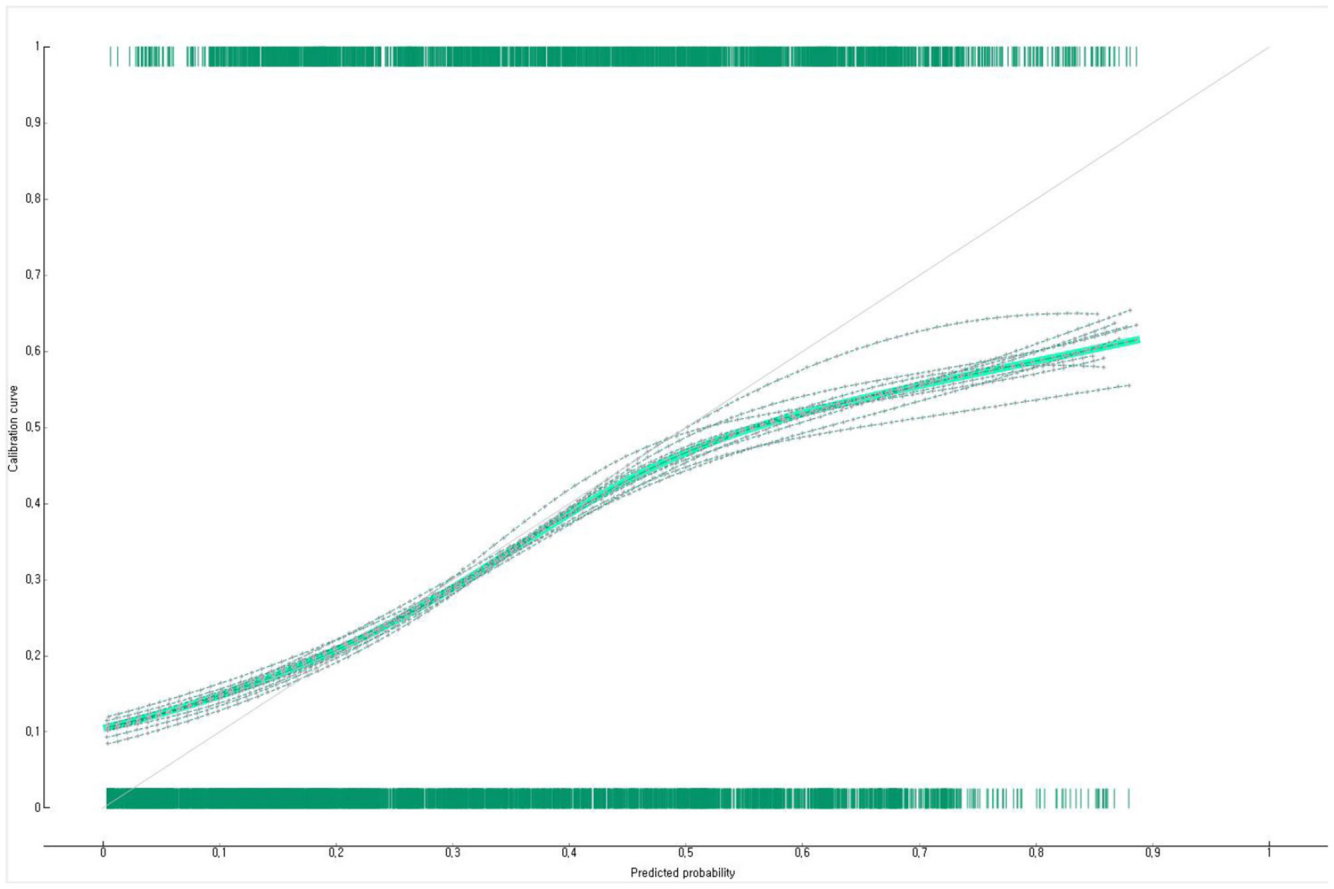
Calibration plot of the Bayesian nomogram for predicting the depressive disorders of female older adults living alone.

The prediction performance of the developed Bayesian nomogram was validated using classification accuracy, AUC, F-1 score, precision, recall, and calibration plot. The results of LOOCV showed that the AUC (Figure 8), classification accuracy (Figure 9), F-score (Figure 10), precision, and recall of the nomogram developed in this study were 0.77, 0.73, 0.71, 0.71, and 0.73, respectively. This study compared the prediction probability and observation probability of a group with depressive disorders and a group without depressive disorders using calibration plot and the chi-square test (Figure 11) to find that there was no significant difference between them (*P* = 0.583).

## Discussion

This study identified factors related to the depressive disorders of female older adults living alone using stacking ensemble machine and confirmed that stress perception, subjective health, n-6 fatty acids, n-3 fatty acids, mean sitting hours per day, and mean sleeping hours per day were key risk factors related to the depressive disorders of female older adults living alone.

It is generally believed that the prevalence of depressive disorders increases when the older adult population perceives stress more (28, 29), is less frequently involved in physical activity (30), has poorer subjective health (31, 32), and experiences sleep disturbance more (33). Nevertheless, there was a limitation in identifying multiple risk factors for depressive disorders because most previous studies (28–33) that explored risk factors for depressive disorders did not examine factors related to depressive disorders while distinguishing older adults living family and those living alone and were limited to identifying individual risk factors using regression analysis. This study evaluated multiple risk factors for depressive disorders in female older adults living alone by developing the Bayesian nomogram. The results showed that female older adults living alone would have a very high-risk probability (96%) of depressive disorders when they perceived stress a lot, perceived their subjective health as poor, slept <5 h per day on average, sat 12.5 h or more per day on average, ate <2.29 g of N6 fatty acid per day, and ate <0.39 of N3 fatty acid per day. Therefore, it is necessary to find the high-risk group of depression with these multiple risk factors among female older adults living in the community and continuously monitor them at the community level to prevent depressive disorders.

Older adults have a strong tendency to complain of stress or physical (abnormal) symptoms first rather than directly complaining about depressive symptoms to their family members or neighbors (34). If the symptoms perceived by older adults belonging to a high-risk group for depression are neglected, the depressive symptoms may be worsened, leading to suicide attempts in extreme cases (35). In particular, neglecting the chronic depressive symptoms of older adults can be a major cause of suicidal thoughts or suicide attempts (36). It is necessary to screen high-risk groups of depression early and to provide them with emotional and psychological therapy because female older adults living alone may experience family separation and domestic abuse after bereavement (37). Nevertheless, only a few studies have identified multiple risk factors for predicting the depressive disorders of female older adults living alone. This study proposed a stacking ensemble machine approach and a Bayesian nomogram as methods to identify multiple risk factors for a disease. Based on the results of this study, it is required to evaluate the multiple risk factors for depression including various measurable factors such as social support.

The advantages of this study were that this study explored the risk factors of depressive disorders from various aspects including sociodemographic factors, health habits, health status, and nutritional factors by using epidemiological data representing South Korean older adults and this study developed a nomogram allowing people to visually and easily identify high-risk groups of depressive disorders. The limitations of this study are as follows. First, this study treated older adults living alone as a single group without classifying them into different types according to causes. However, older adults living alone can be divided into those living alone due to unavoidable circumstances such as bereavement and those living alone due to divorce or separation. These two groups may have different depressive characteristics. Future studies are necessary to identify risk factors for depressive disorders according to the type of older adults living alone. Second, this study could not identify the severity or type of depressive symptoms because this study analyzed the prevalence of depressive disorders in older adults living in the community based on the depression screening test, mainly used in epidemiological investigations. Follow-up studies are needed to classify the types of depressive disorders using medical diagnosis and to explore risk factors according to depression type based on this. Third, although depressive disorders can be affected by social networks such as family relationships, friendship, and social support, this did not evaluate the social networks of older adults living alone. Fourth, the food intake frequency survey is based on the subject's subjective memory, and this method has a possibility of a recall bias. Fifth, this study used the SMOTE, a method widely used to resolve data imbalance. Although SMOTE has the advantage that it does not lose information, it may be disadvantageous in predicting the data of new cases because it reflects only the characteristics between the minority data in the modeling set. Therefore, future studies are required to process imbalance data with improved SMOTE, such as adaptive synthetic sampling, when analyzing data with severe imbalance. Sixth, since it is a cross-sectional study, even risk factors for depression cannot be understood as a causal relationship. Additional longitudinal studies are required to prove causality.

## Conclusions

The results of this study implied that it would be necessary to continuously evaluate complex risk factors such as stress perception, subjective health, n-6 fatty acids, n-3 fatty acids, mean daily sitting hours, and mean daily sleeping hours for detecting the depressive symptoms of female older adults living alone in the community as soon as possible. It is difficult for patients to recognize the symptoms of senile depression in the early stage because these symptoms are mild. To make it worse, older adults living alone rarely visit a doctor's office due to depressive symptoms actively, or they tend to visit a doctor's office after depressive symptoms are already severe. Therefore, the Bayesian nomogram developed in this study can be usefully used to detect and manage the depressive disorders of older adults living alone in primary care at an early stage. Furthermore, it is required to continuously manage high-risk groups at the community level as well as to discover the high-risk groups of depression for older adults living alone based on multiple risk factors.

## Data Availability Statement

Restrictions apply to the availability of these data. Data was obtained from Korea Institute for Health and Social Affairs and are available (from the Korea Institute for Health and Social Affairs/https://www.kihasa.re.kr/en) with the permission of Korea Institute for Health and Social Affairs. Requests to access these datasets should be directed to the Korea Institute for Health and Social Affairs/https://www.kihasa.re.kr/en.

## Ethics Statement

The study was conducted according to the guidelines of the Declaration of Helsinki, and approved by the Institutional Review Board (or Ethics Committee) of University (Protocol Code 20180042 and date: 2018.07.01). The patients/participants provided their written informed consent to participate in this study.

## Author Contributions

HB designed the paper, was involved in study data interpretation, preformed the statistical analysis, and assisted with writing the article.

## Funding

This research was supported by Basic Science Research Program through the National Research Foundation of Korea (NRF) funded by the Ministry of Education (NRF-2018R1D1A1B07041091, NRF-2021S1A5A8062526).

## Conflict of Interest

The author declares that the research was conducted in the absence of any commercial or financial relationships that could be construed as a potential conflict of interest.

## Publisher's Note

All claims expressed in this article are solely those of the authors and do not necessarily represent those of their affiliated organizations, or those of the publisher, the editors and the reviewers. Any product that may be evaluated in this article, or claim that may be made by its manufacturer, is not guaranteed or endorsed by the publisher.

## References

[1] Kim YS Han SH Lee JM Shin G Choi JK Park JM. Senior friendly hospital: a new paradigm for the hospital-based care of the elderly. Kor J Clin Geriatr. (2017) 18:8–14. 10.15656/kjcg.2017.18.1.8

[2] Statistics Korea. Estimation of Future Household. 2017-2047. Daejeon: Statistics Korea (2019).

[3] Han D. Best practices of active aging as a community model in Korea. Innov Aging. (2017) 1:686. 10.1093/geroni/igx004.2450

[4] Joo CL Park JJ Kim A Park NL Lim J Park HS. Health behaviors and lifestyle patterns of elderly living alone in Korea. Korean J Fam Pract. (2019) 9:247–53. 10.21215/kjfp.2019.9.3.24732244453

[5] Statistics Korea. Results of Future Population Projections. Seoul: Statistics Korea (2020).

[6] Korea Institute for Health and Social Affairs. Survey of the Elderly. Sejong: Ministry of Health and Welfare (2017).

[7] Health Insurance Review and Assessment Service. Depression Statistics. Wonju: Health Insurance Review and Assessment Service (2019).

[8] Girgus JS Yang K Ferri CV. The gender difference in depression: are elderly women at greater risk for depression than elderly men? Geriatrics. (2017) 2:35. 10.3390/geriatrics2040035 31011045 PMC6371140

[9] Chou KL Ho AH Chi I. Living alone and depression in Chinese older adults. Aging Ment Health. (2006) 10:583–91. 10.1080/13607860600641150 17050087

[10] Förster F Luppa M Pabst A Heser K Kleineidam L Fuchs A. The role of social isolation and the development of depression. A comparison of the widowed and married oldest old in Germany. Int J Environ Res Public Health. (2021) 18:698. 10.3390/ijerph18136986 34210083 PMC8297151

[11] Kim NC Yang S. Physical health status and depression of a community-dwelling elderly group. J Korean Acad Nurs Adm. (2001) 31:1012–20. 10.4040/jkan.2001.31.6.1012

[12] Allan J Dixon A. Older women's experiences of depression: a hermeneutic phenomenological study. J Psychiatr Ment Health Nurs. (2009) 16:865–73. 10.1111/j.1365-2850.2009.01465.x 19930360

[13] Jayawickreme N Atefi E Jayawickreme E Qin J Gandomi AH. Association rule learning is an easy and efficient method for identifying profiles of traumas and stressors that predict psychopathology in disaster survivors: the example of Sri Lanka. Int J Environ Res Public Health. (2020) 17:2850. 10.3390/ijerph17082850 32326220 PMC7215723

[14] Byeon H. Exploring factors for predicting anxiety disorders of the elderly living alone in South Korea using interpretable machine learning: a population-based study. Int J Environ Res Public Health. (2021) 18:7625. 10.3390/ijerph18147625 34300076 PMC8305562

[15] Kandel I Castelli M Popovič A. Comparing stacking ensemble techniques to improve musculoskeletal fracture image classification. J Imaging. (2021) 7:100. 10.3390/jimaging7060100 PMC8321344 39080888

[16] Byeon H. Development of a nomogram for predicting depression in the elderly using Patient Health Questionnaire-9 among a nationwide sample of Korean elderly. J Pers Med. (2021) 11:645. 10.3390/jpm11070645 34357112 PMC8303561

[17] Park SJ Choi HR Choi JH Kim KW Hong JP. Reliability and validity of the Korean version of the Patient Health Questionnaire-9 (PHQ-9). Anxiety Mood. (2010) 6:119–24.

[18] Spitzer RL Kroenke K Wiliams JB. Patient Health Questionnaire primary care study group. Validation and utility of a self-report version of PRIME-MD: the PHQ primary care study. JAMA. (1999) 282:1737–44. 10.1001/jama.282.18.1737 10568646

[19] Werneck AO Stubbs B Szwarcwald CL Silva DR. Independent relationships between different domains of physical activity and depressive symptoms among 60,202 Brazilian adults. Gen Hosp Psychiatry. (2020) 64:26–32. 10.1016/j.genhosppsych.2020.01.007 32086172

[20] Lee H Lee JA Brar JS Rush EB Jolley CJ. Physical activity and depressive symptoms in older adults. Geriatr Nurs. (2014) 35:37–41. 10.1016/j.gerinurse.2013.09.005 24144579

[21] Choi HS Choi JH Park KH Joo KJ Ga H Koh HJ. Standardization of the Korean version of Patient Health Questionnaire-9 as a screening instrument for major depressive disorder. Korean J Fam Med. (2007) 28:114–9.

[22] Ye S Zhang H Shi F Guo J Wang S Zhang B. Ensemble learning to improve the prediction of fetal macrosomia and Large-for-Gestational Age. J Clin Med. (2020) 9:380. 10.3390/jcm9020380 32023935 PMC7074295

[23] Cho G Yim J Choi Y Ko J Lee SH. Review of machine learning algorithms for diagnosing mental illness. Psychiatry Investig. (2019) 16:262–9. 10.30773/pi.2018.12.21.2 30947496 PMC6504772

[24] Sarica A Cerasa A Quattrone A. Random forest algorithm for the classification of neuroimaging data in Alzheimer's disease: a systematic review. Front Aging Neurosci. (2017) 9:329. 10.3389/fnagi.2017.00329 29056906 PMC5635046

[25] Feng L Li Y Wang Y Du Q. Estimating hourly and continuous ground-level PM2. 5 concentrations using an ensemble learning algorithm: the ST-stacking model. Atmos Environ. (2020) 223:117242. 10.1016/j.atmosenv.2019.11724226780051

[26] Byeon H. Exploring factors associated with the social discrimination experience of children from multicultural families in South Korea by using stacking with non-linear algorithm. Int J Adv Comput Sci Appl. (2021) 12:125thm. 10.14569/IJACSA.2021.0120516

[27] MoŽina M Demšar J Kattan M Zupan B. Nomograms for visualization of naive bayesian classifier. In European conference on principles of data mining and knowledge discovery. Knowl Discov Datab. (2004) 3202:337–48. 10.1007/978-3-540-30116-5_32

[28] Tsai AC Chi SH Wang JY. Association of perceived stress with depressive symptoms in older Taiwanese: results of a population-based study. Geriatr Gerontol Int. (2015) 15:535–43. 10.1111/ggi.12307 24851696

[29] Kim B. Factors influencing depressive symptoms in the elderly: using the 7th Korea National Health and Nutrition Examination Survey (KNHANES VII-1). J Health Info Stat. (2020) 45:165–72. 10.21032/jhis.2020.45.2.165

[30] Zhang S Xiang K Li S Pan HF. Physical activity and depression in older adults: the knowns and unknowns. Psychiatry Res. (2021) 297:113738. 10.1016/j.psychres.2021.113738 33515871

[31] Pinquart M. Correlates of subjective health in older adults: a meta-analysis. Psychol Aging. (2001) 16:414–26. 10.1037/0882-7974.16.3.414 11554520

[32] Oh CS. The effects of the elderly's subjective health perceptions and quality of life on their depression and suicide ideation. Korean J heal serv Manag. (2012) 6:179–91. 10.12811/kshsm.2012.6.2.179

[33] Yu J Rawtaer I Fam J Jiang MJ Feng L Kua EH. Sleep correlates of depression and anxiety in an elderly Asian population. Psychogeriatr. (2016) 16:191–5. 10.1111/psyg.12138 26179204

[34] Kok RM Reynolds CF III. Management of depression in older adults: a review. JAMA. (2017) 317:2114–22. 10.1001/jama.2017.5706 28535241

[35] Conejero I Olié E Courtet P Calati R. Suicide in older adults: current perspectives. Clin Interv Aging. (2018) 13:691–9. 10.2147/CIA.S130670 29719381 PMC5916258

[36] Minayo MC Cavalcante FG. Suicide in elderly people: a literature review. Rev Saude Publica. (2010) 44:750–7. 10.1590/S0034-89102010000400020 20676565

[37] Lee J Lee C Min J Kang DW Kim JY Yang HI. Development of the Korean Global Physical Activity Questionnaire: reliability and validity study. Glob Health Promot. (2020) 27:44–55. 10.1177/1757975919854301 31375056

